# Recent Advances in the Reverse Water–Gas Conversion Reaction

**DOI:** 10.3390/molecules28227657

**Published:** 2023-11-18

**Authors:** Changjian Zhou, Jiahao Zhang, Yuqing Fu, Hui Dai

**Affiliations:** 1 School of Chemistry and Chemical Engineering, Yancheng Institute of Technology, Yancheng 224051, China; zcj@ycit.cn (C.Z.);; 2 College of Materials and Chemistry & Chemical Engineering, Chengdu University of Technology, Chengdu 610059, China

**Keywords:** CO_2_ conversion, CO, reverse water–gas conversion reaction, catalyst design

## Abstract

The increase in carbon dioxide emissions has significantly impacted human society and the global environment. As carbon dioxide is the most abundant and cheap C1 resource, the conversion and utilization of carbon dioxide have received extensive attention from researchers. Among the many carbon dioxide conversion and utilization methods, the reverse water–gas conversion (RWGS) reaction is considered one of the most effective. This review discusses the research progress made in RWGS with various heterogeneous metal catalyst types, covering topics such as catalyst performance, thermodynamic analysis, kinetics and reaction mechanisms, and catalyst design and preparation, and suggests future research on RWGS heterogeneous catalysts.

## 1. Introduction

With the development of industry and human activities, the concentration of carbon dioxide (CO_2_) in the global atmosphere is increasing yearly. As the main component of greenhouse gases, the CO_2_ concentration in the atmosphere continues to rise, causing a series of severe environmental problems such as climate warming, glacier melting, and ocean acidification, seriously threatening the living environment of human beings [1].

At present, fossil fuels are still the main mode for humans to obtain primary energy, and forms of renewable energy, such as solar and wind energy, are still in the development stage and cannot yet fully replace fossil fuels. CO_2_ is an abundant, inexpensive, non-toxic, non-flammable, and renewable single-carbon structural unit that can be used as a good carbon source.

In recent years, in order to cope with the negative impact of CO_2_, in addition to developing new energy sources, carbon capture and storage (CCS) and carbon capture and utilization (CCU) technologies have attracted widespread attention due to their high efficiency and easy application in capturing large amounts of CO_2_ [2]. CCS compresses CO_2_ from the source into high-density CO_2_, making it suitable for long-term transportation, storage, and monitoring, but the potential risk of leakage and continuous on-site monitoring are the main disadvantages related to this technology. Meanwhile, CCU uses different strategies to convert carbon dioxide into useful chemical products such as fuels (such as methanol) and materials (polymers) [3,4]. CCU is more useful than CCS because value-added products can be obtained using the captured CO_2_. In addition, CCU also offers no possibility of carbon dioxide leakage, which makes it promising in terms of sustainability and environmental friendliness [5]. CO_2_ utilization modes mainly include biological utilization, mineralization utilization, chemical synthesis, etc. In many CO_2_ utilization technologies, CO_2_ produces a variety of high−value basic chemicals through catalytic conversion, a process that has been widely studied by researchers. CO_2_ catalytic conversion technology mainly includes thermal catalysis [6,7,8,9], photocatalysis [10,11], electrocatalysis [12,13], and so on. The research on CO_2_ catalytic conversion mainly focuses on CO_2_ catalytic hydrogenation.

CO_2_ catalytic conversion research focuses on CO_2_ catalytic hydrogenation, a very green and environmentally friendly method for converting carbon dioxide into high-value substances such as CO, methanol, olefins, and alkanes [14,15]. According to the reaction pathway, the catalytic hydrogenation of CO_2_ mainly generates CO [16,17], methane [18,19], and hydrocarbons [20,21].

In summary, whether from the perspective of protecting the environment or CO_2_ utilization, the catalytic hydrogenation of CO_2_ is a method with broad application prospects to reduce the greenhouse effect and provide energy resources. However, CO_2_ has a very stable C=O bond and is thermodynamically difficult to activate [22]. Researchers have tried to reduce the activation energy of the CO_2_ reduction reaction by adding catalysts to achieve the goal of efficient CO_2_ conversion. Therefore, preparing simple and easily synthesized high-activity catalysts to convert carbon dioxide into high−value−added products is significant for improving the natural environment and addressing the energy demand.

CO_2_ catalytic hydrogenation to CO is known as the reverse water–gas conversion (RWGS) reaction. The CO produced via this reaction can be used as a syngas, often used to synthesize methanol, higher alcohols, or Fischer–Tropsch fuels. This method is expected to be an effective way to achieve carbon neutrality and cycling and will significantly impact the environment and the future energy mix.

RWGS mainly converts the greenhouse gas CO_2_ into CO. However, this reaction also produces the byproduct CH_4_. Therefore, developing an RWGS reaction catalyst with high CO selectivity is necessary to convert CO_2_ into CO in the RWGS reaction. The RWGS reaction is a typical endothermic reaction. From a thermodynamic point of view, since the RWGS reaction is endothermic, increasing the temperature during the reaction is more conducive to the equilibrium moving in a positive direction in the RWGS reaction. From a kinetic point of view, it has a higher reaction rate at a high temperature. Combined with thermodynamics and kinetics, the RWGS reaction is a process with high energy consumption [23]. At the same time, a high temperature will also lead to catalyst sintering, carbon deposition, and deactivation. Therefore, developing low-temperature, high−activity, and high-selectivity RWGS reaction catalysts has become the focus of current research, and many reviews of this work have been published [1,24,25,26,27,28,29]. Here, we review the research progress made in RWGS with different kinds of heterogeneous metal catalysts, including catalyst design and preparation, catalyst performance, thermodynamic analysis, kinetics and mechanism of reaction, etc., and predict future research on RWGS heterogeneous catalysts.

## 2. Results and Discussion

### 2.1. Reaction Mechanism of the Reverse Water–Gas Conversion Reaction

**Figure 1:**
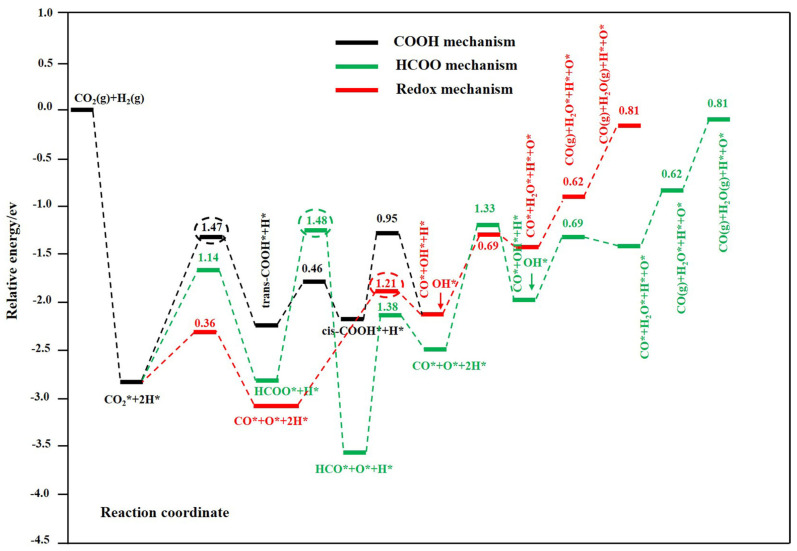
Potential energy reaction diagram of Cu@Mo_2_C(001) with preabsorbed OH* species. The associated mechanism via COOH is shown by the black line, which via HCOO is shown by the green line, and the redox mechanism is shown by the red line. The redox mechanism is the most favorable pathway at 300–600 °C. Calculated potential energy profile of the most favorable redox (red line), HCOO (green line), and COOH (black line ) mechanism for the RWGS reaction on the Cu@Mo_2_C(001) surface. The numbers in the figure are the activation barriers of the elementary steps. The numbers in red, green, and black circles are the activation barriers of the rate–limiting steps in the redox, HCOO, and COOH pathways, respectively. Reprinted with permission from ref [30]. Copyright American Chemical Society 2019.

**Figure 2:**
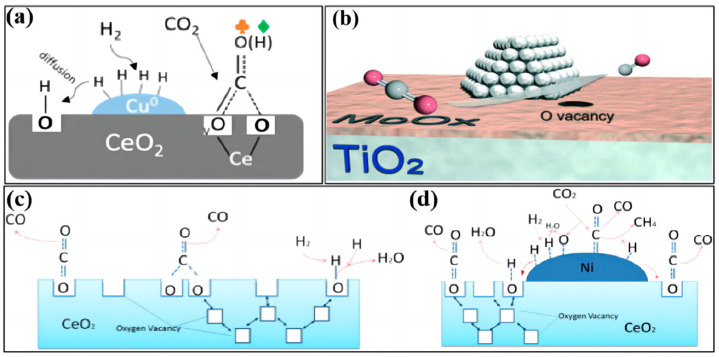
The redox reaction mechanism of the RWGS reaction over (**a**) Cu−CeO_2_ (reprinted with permission from [31]. Copyright American Chemical Society, 2018), (**b**) Pt/MoO_x_−TiO_2_ (reprinted with permission from [32]. Copyright Royal Society of Chemistry, 2021), (**c**) ceria nanocubes (reprinted with permission from [33]. Copyright Elsevier, 2016), and (**d**) Ni−CeO_2_ nanocubes (reprinted with permission from [33]. Copyright Elsevier, 2016).

**Figure 3:**
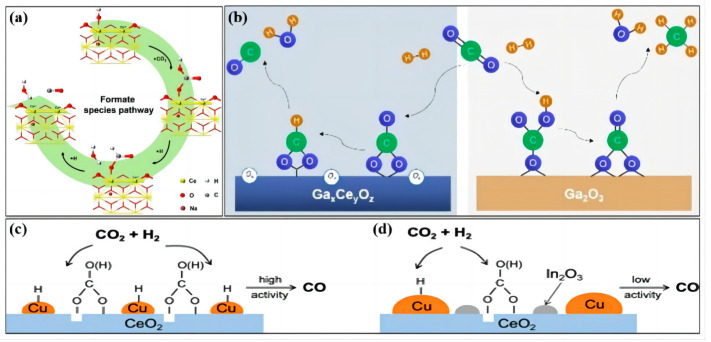
The associative mechanism of the RWGS reaction over (**a**) LN−CeO_2_ (reprinted with permission from [34]. Copyright Elsevier, 2022), (**b**) Ga_2_O_3_−CeO_2_ (reprinted with permission from [9]. Copyright Wiley, 2022), (**c**) Cu−CeO_2_ (reprinted with permission from [35]. Copyright American Chemical Society, 2022), and (**d**) Cu_5_In_5_−CeO_2_ (reprinted with permission from [35]. Copyright American Chemical Society, 2022).

The RWGS reaction has unique advantages as one of the ways to utilize CO_2_. In order to design a reasonable catalyst, it is important to understand the mechanism of CO formation in the CO_2_ hydrogenation reaction in detail. The RWGS reaction’s mechanism has always been a topic of debate among researchers, and existing researchers have proposed two RWGS reaction mechanisms, namely a redox mechanism (Figure 1 and Figure 2) and an intermediate species decomposition mechanism (Figure 1 and Figure 3). Which reaction mechanism is followed depends on the type of catalyst or reaction conditions used. The two mechanisms reported in the current literature are still controversial. It is necessary to study the reaction mechanism of various catalyst types for catalyst development.

(1) Redox mechanism

This mechanism mainly refers to the continuous oxidation and reduction of the active species in the catalyst in the CO_2_ and H_2_ atmosphere during the RWGS reaction to sustain the catalytic reaction. CO_2_ is first adsorbed on the catalyst, combining with the active site on the catalyst surface after dissociation to produce CO. The generated active oxygen substances exist on the active site of the catalyst and then continue to reduce the catalyst to generate H_2_O and release the surface active site to continue participating in the next round of the reaction. H_2_ is not directly involved in the synthesis of intermediate species. In this process, CO_2_ dissociation is the rate-limiting step, and the adsorption and desorption steps of CO and H_2_O can be ignored. The basic reactions of the surface redox mechanism are as follows (X indicates an empty active site with no interaction with the adsorbent):

CO_2_ + X = CO_2_·X

CO_2_·X = CO + O·X

H_2_ + 2 X = 2 H·X

2 H·X + O·X = H_2_O + 3 X

Gines and co-workers [36] prepared two catalysts, CuO/ZnO/Al_2_O_3_ and CuO/Al_2_O_3_, using the co-precipitation method. The mechanism of surface redox was proven via a kinetic experiment based on the RWGS reaction. According to the kinetic experiment results, it was found that the reaction rate of CO_2_ is controlled by dissociative adsorption. The chromatographic analysis showed that only CO was formed when CO_2_/N_2_ was inserted, which indicated that CO_2_ was dissociated from the active site on the catalyst surface to form CO. In the H_2_ atmosphere, only H_2_O was produced, indicating that H_2_ combines with active oxygen species on the catalyst surface to form H_2_O. Wang et al. [37] found that the oxygen vacancies that form on the surface of the pre−reduced Au/CeO_2_ catalyst are oxidized by CO_2_ to produce CO. This process corresponds to the redox mechanism of RWGS. The author also loaded Au onto different carriers and found through a pulse experiment that the amount of active oxygen on the surface of CeO_2_ carriers was significantly higher than that on TiO_2_ carriers. This is because CeO_2_ has a good oxygen storage and supply capacity and can assist Au atoms to complete the removal and production of surface-reactive oxygen species.

(2) Decomposition mechanism of intermediate species

In the RWGS reaction, the decomposition mechanism of adsorbed intermediate species refers to CO_2_ and H_2_ on the catalyst, first being activated to form intermediate species and then decomposed into CO and H_2_O. In recent years, in most of the literature on RWGS reactions, formates, carbonates, and carbonyl groups were the main intermediate species formed and are considered the key steps for further CO generation.

It has been found in many research reports that there are different intermediates in RWGS reactions with different catalysts, and factors such as the properties of catalysts, the different interactions between metals and carriers, and the differences in reaction conditions lead to the complexity of the decomposition mechanism of intermediate species in RWGS reactions.

Liu et al. [38] conducted a CO_2_ dissociation experiment to explore the reaction pathway. Combined with the CO_2_ dissociation experiment results and temperature−programmed surface reaction, CO_2_ activation can be carried out through relevant intermediate pathways. In order to further explore the active intermediate, in situ diffuse infrared Fourier transform spectroscopy (DRIFTS) is performed. After the injection of CO_2_ and H_2_ into the activated 15CuCe catalyst, the formate signal can be detected in addition to the carbonate signal. In the presence of H_2_, the Cu atom captures the H_2_ molecule, breaks the H–H bond, and then transfers the H atom to the CO_2_. Formate structures are formed with the formation of C–H bonds, and these structures are manifested in the intermediates IMA3, IMA3−I, IMA4, and IMA4−I. Based on the above experimental results and density functional theory (DFT) calculation, the authors further concluded that the reaction involved the association mechanism, and the surface formates and carboxylic acid species may be important reaction intermediates. In the reaction process, a large number of surface oxygen vacancies are generated in situ and recycled, forming a synergistic catalytic effect with copper clusters, promoting the activation of CO_2_ and the formation of active intermediates. The copper clusters and abundant oxygen vacancies in the 15CuCe catalyst undoubtedly create more oxygen−vacancy−active interfaces of metal clusters.

Deng et al. [39] explored the mechanism of a Cu−based slag-based geopolymer microsphere (SGS) catalyst in the RWGS reaction and conducted in situ DRIFTS experiments to observe intermediates generated during the catalytic process. The characteristic bands of bicarbonate and carbonate appeared. The formate band appeared at about 150 °C and then gradually weakened above 200 °C, indicating that formate species formed at low temperatures. The SGS is an alkaline carrier with many surface OH groups. With the increase in temperature, the OH band gradually weakened, indicating that the surface OHs participated in the catalytic process. According to DRIFTS experiment results, we observe that a gas-phase CO band appears above 250 °C, and this band becomes stronger with increasing temperature. However, no formate bands were observed above 250 °C, so it can be inferred that formate species are not intermediates in CO production. Carboxylate bands were found at 1260 cm^−1^ and 1280 cm^−1^, so it was determined that the reaction mechanism of a Cu/SGS catalyst in the RWGS reaction is similar to the carboxylate pathway, and *COOH is the intermediate of CO_2_ to CO. Combined with the results of CO_2_ TPD, it can be determined that the formation of *COOH comes from the adsorption and activation of CO_2_ by the abundant OH groups on the SGS carrier, where OHs on the surface of SGS react with CO_3_^2−^ to form HCO_3_^−^. H_2_ is adsorbed on Cu to form H*, and the HCO_3_^−^ substance reacts with H* on the surface of Cu to form *COOH. In addition, *COOH reacts with H* on the Cu surface to form CO* and OH*, and then CO and H_2_O dissociate from the Cu surface.

### 2.2. Overview of Catalysts of Different Systems in the Reverse Water–Gas Conversion Reaction

In industrial production, stable catalysts with high activity and selectivity need to be considered at low temperatures. However, designing a catalyst that meets all these criteria simultaneously is challenging. The current catalysts used for RWGS are mainly divided into precious metal catalysts and non-precious metal catalysts. Some representative research results are listed in Table 1.

### 2.3. Precious Metal Catalysts

Precious metal-supported catalysts are the most common catalysts for RWGS. The main active species are Pt, Pd, Au, Ir, Rh, Ru, etc. The main carriers are SiO_2_, CeO_2_, TiO_2_, and Al_2_O_3_, on which precious metals promote the dissociation of H_2_, while oxide carriers facilitate the breakage of the C=O bond in CO_2_. The dispersion and chemical state of precious metal nanoparticles are the key factors affecting catalyst performance and significantly impact the adsorption behavior of reactants on the catalyst and the subsequent intermediate species transformation [40].

#### 2.3.1. Pt–Based Catalysts

Supported Pt catalysts [32,41,42,43,60,61,62,63] are widely used in RWGS reactions due to their excellent H_2_ dissociation and hydrogenation activity.

Chen et al. [42] found that the activation energy of CO production on a Pt/CeO_2_ catalyst was significantly lower than that on a pure CeO_2_ catalyst. The calculated TOF values were roughly the same across these Pt/CeO_2_ catalysts, indicating that the RWGS reaction was insensitive to the size of the anchored Pt particles and the primary crystallinity of the CeO_2_ carrier. The results of temperature-programmed reduction (TPR) and X-ray photoelectron spectra (XPS) showed that with the addition of Pt, the reducibility of the CeO_2_ carrier was enhanced, oxygen vacancies were more easily generated, and CO_2_ activation was accelerated. In addition, using in situ Fourier transform infrared spectroscopy (FT−IR) and temperature-programmed surface reaction-mass spectrometry (TPSR−MS) experiments, we found that redox and dissociation mechanisms co-exist in RWGS reactions on Pt/CeO_2_ catalysts. CO_2_ molecules adsorbed at the Ce^3+^ active site cannot directly generate CO, the same as the previous redox mechanism.

Liu et al. [43] prepared Pt-Re/SiO_2_ catalysts with different Re contents using co-impregnation and tested the RWGS reaction. The characterization results showed that the oxygenophilic ReO_x_ (0 ≤ x ≤ 3.5) near the Pt particles modified the Pt surface through partial covering and electron interaction, resulting in decreased CO adsorption sites and adsorption strength. At 400 °C, the turnover frequency of the optimal PT−Re/SiO_2_ catalyst is 3.9 times higher than that of Pt/SiO_2_, and the apparent activation energy is reduced. The CO selectivity on Pt−Re/SiO_2_ remains above 96.2% compared to Re/SiO_2_, which produces large amounts of CH_4_. The reaction order analysis showed that Pt promoted H_2_ activation, while oxyphilic ReOx enhanced CO_2_ adsorption and activation. The peripheral sites of the Pt/ReO_x_ interface have C–O cleavage properties, which can synergistically increase RWGS activity and inhibit the production of CH_4_.

To improve the potential of plasma effects on H−doped WO_y_, Ge et al. [62] reported that Mo−doped Pt/WO_y_ (Pt/MoWO_y_) significantly increases the concentration of dopant (H^+^) and oxygen vacancies in Pt/H_x_MoWO_y_ during H_2_ reduction, promoting the photothermal hydrogenation of CO_2_ to CO. The developed Pt/H_x_MoWO_y_ showed excellent catalytic performance (3.1 mmol·h^−1^·g^−1^) in the photothermal RWGS reaction at 140 °C, which was superior to the undoped Pt/H_x_WO_y_ (1.02 mmol·h^−1^·g^−1^). The experiment and comprehensive analysis show that the abundant surface-free electrons and oxygen vacancies (VOs) in Pt/H_x_MoWO_y_ are the reasons for the effective CO_2_ adsorption and transfer. The characterization of catalysts revealed a reversible redox of Mo and W atoms during RWGS reactions, confirming that oxygen vacancies between Mo and W atoms in Pt/H_x_MoWO_y_ act as active sites. Pt nanoparticles activate H_2_ to regenerate oxygen vacancies. In addition, density functional theory calculations show that Mo doping significantly reduces the energy barrier of oxygen vacancy formation in WO_y_ during H_2_ reduction.

He et al. [63] synthesized a single–atom catalyst, Pt_1_/SiC, in which the Pt particles are uniformly dispersed on SiC and applied it in the conversion of CO_2_ via the reverse water–gas shift reaction, exhibiting 100% selectively and 54% CO_2_ conversion at 900 °C with a H_2_/CO_2_ ratio of 1:1. It was found that in the first few hours, the Pt_1_/SiC catalyst showed excellent stability with negligible decline in activity. However, over time, Pt_1_/SiC was gradually deactivated. After the reaction lasted 10 h, the CO_2_ conversion rate remained relatively stable at about 50%. The authors attributed the decrease in catalyst activity to two factors: the poisoning of the Pt_1_/SiC catalyst caused by CO molecules and the reduction of a small amount of Pt_1_/SiC.

#### 2.3.2. Pd–Based Catalysts

Many researchers have focused on the design, preparation, and mechanism of Pd catalysts [44,64,65,66,67,68,69,70,71] based on their catalytic performance in the RGWS reaction. Here are some selected examples discussed in detail.

Nelson et al. [64] dispersed Pd on a TiO_2_ carrier. The results show that in the RWGS reaction, Pd is mainly dispersed on titanium dioxide in the form of isolated atoms. Achieving atomic dispersion requires artificially increasing the absolute surface area of titanium dioxide by an order of magnitude, which can be achieved by physically mixing the catalyst Pd/TiO_2_ with pure titanium dioxide prior to the RWGS reaction. Kinetic analysis, infrared spectroscopy, X−ray absorption spectroscopy, and scanning electron microscopy showed that the RWGS activity of the Pd/TiO_2_−0.01 catalyst was very good within 92 h after the in situ dispersion of Pd atoms. The thermodynamic stability of Pd under high-temperature RWGS reaction conditions is related to the Pd–Ti coordination, which is related to the formation of oxygen vacancies and the artificial increase in titanium dioxide’s surface area.

Onodera et al. [65] discovered that, up to 300 °C, Au−doped Pt/CeO_2_ catalysts, which were created using the electroless plating method, demonstrated comparable CO_2_ conversion activities to the Pt/CeO_2_ catalyst. Furthermore, above 250 °C, the CO selectivity of the Au−doped Pt/CeO_2_ catalysts was significantly higher than that of the Pt/CeO_2_ catalyst because the addition of the proper amount of Au to the Pt/CeO_2_ catalyst created a favorable density of state for the RWGS reaction. However, a higher density of state leads to a greater electron donation from the Au-doped Pt/CeO_2_ catalyst to an antibonding orbital of CO_2_, which weakens the O−C−O bonds and accelerates the methanation reaction. It is hypothesized that by managing the density of the state close to the Fermi level, the Pt/CeO_2_ catalyst’s increased CO selectivity could be realized.

RWGS reactivity is enhanced in real reactions by the dual effect of Pd_1_−FeO_x_ single−atom catalysts (SACs), as Du et al. reported [66]. Unexpectedly, Pd SAs not only offer single active sites but also dynamically carburize the FeO_x_ surface over an extended reaction period. This results in exceptional RWGS activity (42.0 mmol CO·g_cat_^−1^·h^−1^) and CO selectivity (>98%), surpassing the results of other documented Fe–based catalysts. The results of this dynamic carburization show that the atmospheric rWGS reaction produces a highly active Fe_5_C_2_ phase with the lowest activation barrier of 27.7 kJ/mol. These results are obtained using cutting–edge characterization techniques. Compared to Pd nanoparticles with a fast FeO_x_ encapsulation that prevents carburization, SACs’ dual effect produces better catalytic performance. The various functions that individual atoms play in actual reactions are revealed by this work, suggesting that SACs have a broader function. During actual reactions, single atoms play a dual role in the formation of a highly active phase of heterogeneous catalysts in addition to serving as independent active sites. It is important for rational designs to take into account both roles.

A series of monodispersed Pd nanoparticles, ranging in size from 2.8 nm to 8.1 nm, were prepared by Yang et al. [67] in a progressive manner. With regard to Pd sizes, the photo−thermal catalytic activity in the RWGS reaction exhibits a volcano−type dependence, with the highest activity occurring at a Pd/TiO_2_ size of 6.3 nm. The tunable−surface electronic properties, such as the quantum size effect and metal–support interaction, are responsible for the size−modulated activity.

#### 2.3.3. Ru−Based Catalysts

Some researchers have also turned their attention to the research of Ru−based catalysts [45,46,72,73,74,75], which we briefly describe below.

Tang et al. [72] prepared an efficient RWGS catalyst by encapsulating a Ru cluster with a size of 1 nm in a hollow silica shell. The space–confined structure prevents the sintering of Ru clusters, and the permeable silica layer allows the diffusion of gaseous reactants and products. This catalyst with reduced particle sizes not only maintains the excellent activity of Ru in the CO_2_ hydrogenation reaction but also exhibits close to 100% CO selectivity and excellent stability at 200–500 °C.

Abdel−Mageed et al. [45] investigated the effect of carrier particle size on the performance of highly active Ru/TiO_2_ catalysts and found that after high−temperature reduction treatment, the selectivity of TiO_2_ particle size can be controlled from 100% methanation to 100% CO. The comprehensive characterization of the catalysts shows that while the reaction behavior changes, their structure, chemical, and electronic properties also change significantly. The chemical modification of the carrier via oxygen-vacancy formation leads to the electronic modification of the Ru centers around the interface, which in turn affects the reaction behavior of these centers in CO_2_ reduction reactions, from methanation to RWGS reactions.

Wu et al. [73] reported effective photocatalysis driven by sunlight over Ru clusters supported by MXene that have strong light–absorption capabilities, high sintering resistance, and increased metal loading. Remarkably, MXene-supported Ru clusters demonstrated superior photothermal catalytic performance compared to silica-supported Ru clusters and MXene−supported Ru nanoparticles by 2 and 81 times, respectively. The superior photothermal characteristics of MXene materials and the robust binding between metal clusters and MXene supports are the cornerstones of our approach. Remarkably, the CO production rate of Ru clusters supported by MXene in the batch reactor was 4.0 mol·g_Ru_^−1^·h^−1^, one of the highest recorded rates for photothermal RWGS catalysts to date. This approach can, in theory, be expanded to metal catalysts with even greater dispersion to promote the development of effective sunlight–driven single-atom catalysis. Future research will thoroughly examine how light affects catalytic reactions, with the goal of adding more photochemical activation to increase photocatalytic efficiency. Their work shows the great potential of metal cluster catalysis powered only by concentrated solar radiation. Developing catalysts with enhanced reactivity and solar power alone presents excellent potential as a low-carbon chemical technology.

Chen et al. [74] examined the mechanisms of the RWGS reaction on Ru adsorption on a three−fold site of the CeO_2_(111) surface using first–principles calculations and microkinetic simulations. Microkinetic simulations covering all elementary reaction steps were used to investigate the effects of temperature on the active site and the preferred reaction mechanism. Ru(OH)_3_−(OH) is the predominant active site at low temperatures, according to the microkinetic simulations. A low barrier to direct CO dissociation is the outcome of this hydroxyl−promoted configuration. RuO_3_ becomes the predominant active site when the temperature rises due to decreased Ru(OH)_3_−(OH) abundance. The Mars–van Krevelen mechanism is the one that is favored in such circumstances. Heat makes forming oxygen vacancies easier, which serve as CO_2_ anchoring sites. When CO_2_ adsorbs, the oxygen vacancies are restored, increasing the CO_2_ molecule’s reactivity and facilitating the simultaneous scission of C−O bonds and CO desorption. Ultimately, their research suggests a temperature-dependent RWGS mechanism and emphasizes the impact of surface hydroxyl and oxygen vacancies on RWGS compared to the Ru/CeO_2_ single−atom catalyst.

#### 2.3.4. Au−Based Catalysts

In addition to the above precious metal catalysts, some Au-based catalysts have also been studied and reported, and the following is a brief introduction.

Abdallah et al. [34] loaded a titanium dioxide and zirconia support with a very low content (<0.1 wt%) of Au, used it for the RWGS reaction, and found that this gold-based catalyst showed high catalytic activity for the RWGS reaction at low temperatures. At 250 °C, the catalytic activity of the Au/TiO_2_ catalyst is nearly 10 times that of Au/ZrO_2_. The in situ infrared DRIFT results show that the formate is the main intermediate species on the Au/ZrO_2_ catalyst, while on the Au/TiO_2_ catalyst, the reaction is carried out via the formation of hydroxyl carbonyl intermediates. The results of scanning transmission electron microscopy (STEM), STEM−EELS (electron−energy−loss spectrometry), XPS, and EPR in situ indicate that the Au–O_v_–Ti^3+^ interface sites are responsible for the excellent activity of Au/TiO_2_.

To bridge the knowledge gap regarding the completeness of the plasma–thermal coupling mechanism for Au/*γ*−Al_2_O_3_, Wang et al. [76] studied the photo−thermocoupled catalytic RWGS reactions and their reaction pathway and plasmonic enhancement mechanism. As per the findings, the total reaction is facilitated by both the formate and carboxyl pathways. A low reaction temperature over small Au NPs is proposed to be mediated primarily by the m−formate pathway. Combining resonance and hot-electron energy-transfer mechanisms, spectro–kinetics, and theoretical computation analyses show that the plasmonic energy preferentially moves to HCOO*. As the rate−determining step (RDS) of the entire RWGS reaction, the plasmonic energy facilitates the dehydration of HCOO* to CO.

#### 2.3.5. Some Other Precious Metal-Based Catalysts

In addition to introducing the above precious metal catalysts, here we also introduce these relatively less reported precious metal catalysts, such as Bi, Pt−Re, Re, Rh, Ir, In, etc.

Kang et al. [77] reported a Bi-based photothermal RWGS catalyst. Using a soft template technique, they were able to create Bi single atoms loaded with CeO_2_ nanosheets that demonstrated exceptional stability and activity for RWGS at 400 °C air corrosion, demonstrating previously unheard-of oxidation resistance. High activity and oxidation resistance for RWGS result from the Bi single atoms’ low energy barrier and their +3 valence state retention during RWGS, as demonstrated via experiments and first−principles calculations. Loading BiO_x_/CeO_2_ into a Ti_2_O_3_−based photothermal system resulted in a CO generation rate of 31.00 mmol g^−1^ h^−1^, four times higher than the state−of−the−art for solar-driven RWGS under 20 sun irradiation units. This was accomplished under three sun units of radiation.

RWGS reaction tests were conducted by Liu [43] on Pt−Re/SiO_2_ catalysts that varied in Re content and were prepared via co–impregnation. The results of the characterization showed that the oxophilic ReO_x_ (0 ≤ x ≤ 3.5) that was close to the Pt particle changed the surface of Pt by partial coverage and electronic interaction, which led to a decrease in the number of sites and a weakening of the strength of CO adsorption. When Pt/Re/SiO_2_ is optimized (Pt/Re = 1.91), the turnover frequency (2.30 s^−1^) is 3.9 times higher at 400 °C than when Pt/SiO_2_ is used under differential conditions, and the apparent activation energy is decreased. Pt–Re/SiO_2_’s CO selectivity maintained >96.2% under integral conditions, unlike Re/SiO_2_, which generated a significant amount of CH_4_. Pt helps to activate H_2_, while ReO_x_, an oxophile, helps to activate and adsorb CO_2_. This was determined using reaction order analysis. The RWGS activity is enhanced while CH_4_ production is inhibited by the perimeter sites at the Pt/ReO_x_ interface that have balanced hydrogenation and C–O cleavage properties.

Yang et al. [78] clearly demonstrated the close relationship between the size effect, the active site, and the reaction mechanism using a special set of well-defined TiO_2_-supported Re catalysts and systematic characterizations. Additionally, a size-dependent, wave−like activity of CO_2_ conversion was found. CO_2_ hydrogenation is regulated by the RWGS reaction in the size range of a single atom to 1.0 nm. As size increases, the turnover frequency of this reaction falls. Nevertheless, CO_2_ methanation takes over as the primary reaction and exhibits a size-dependent performance resembling a volcano for clusters larger than 1 point 0 nm. According to the mechanistic analysis, formate pathways in single-atom catalysts allow the perimeter site to control the RWGS reaction. The edge site in the Re cluster, on the other hand, serves as the active site. Here, CO_2_ undergoes a redox process to reduce CO and then hydrogenates to methane across the edge sites. This finding could contribute to a better mechanistic understanding of structure sensitivity.

In Liu’s work [79], it was successfully constructed to perform the reverse water–gas shift reaction mediated by the diatomic anion Rh^2−^. At room temperature, the production of a gas−phase H_2_O molecule and the ion product [Rh_2_(CO)_ads_]^−^ were clearly identified, and the desorption of CO from [Rh_2_(CO)_ads_]^−^ was the only basic step that needed additional energy to finish the catalysis. Since designing efficient routes to yield H_2_O from CO_2_ and H_2_ is difficult, this experimentally identified Rh^2−^ anion represents the first gas–phase species that can drive the RWGS reaction. Their recently created double-ion trap reactors were used to carry out the reactions, which were then examined using high−precision quantum-chemical calculations, photoelectron spectroscopy, and mass spectrometry. They discovered that the distribution of the final products (D_2_O and Rh_2_CO^−^) and the reactivity were not significantly affected by the order in which the reactants (CO_2_ or D_2_) were fed into the reactor. The essential steps to steer the reaction in the direction of the RWGS were revealed with atomic precision.

In Lu’s work [80], a solvent evaporation self-assembly method was used to build a system of Ir species and *α*−MoC. Because of the synergistic effect of Ir and *α*–MoC, the catalytic performance of the Ir/MoC catalysts for the RWGS reaction was significantly better than that of pure *α*−MoC over a wide temperature range (200–500 °C). At 500 °C, 0.1 MPa, and 300,000 mL·g^−1^·h^−1^, the ideal 0.5% Ir/MoC catalyst produced a 48.4% CO_2_ conversion, similar to the equilibrium conversion of 49.9%. Higher than most previously reported values, the CO selectivity and space–time yield of CO over 0.5%Ir/MoC reached 94.0% and 423.1 umol·g^−1^·s^−1^, respectively. Furthermore, over a 100 h period, 0.5% Ir/MoC maintained its catalytic properties and showed exceptional stability at elevated temperatures. It was shown via several characterization techniques that the Ir species supported on *α*−MoC substrates were widely distributed. The stability of the Ir/MoC catalysts was significantly enhanced by the strong metal–support interaction that took place via electron transfer between Ir and α-MoC. Ir single atoms (Ir_1_) and clusters (Ir_n_) coexisted to form Ir_n_−Ir_1_−C–Mo synergistic sites between Ir and *α*−MoC for the Ir/MoC catalysts with Ir loadings > 0.2% (mass fraction). Comparing the 0.5% Ir/MoC catalyst to the other Ir/MoC catalyst, the number of Ir_1_ species and size of Ir_n_ species were higher and smaller, respectively. The exceptional adsorption and activation of CO_2_ and H_2_ during the RWGS were facilitated by the optimal electron density conferred upon 0.5% Ir/MoC. The RWGS reaction mechanism was found to occur via a formate pathway in in situ diffuse-reflectance infrared Fourier transform spectroscopy experiments. The creation and breakdown of formate intermediates were noticeably aided, even though the Ir_n_−Ir_1_−C−Mo synergistic site formation had no effect on the reaction mechanism. The synergistic effect, therefore, effectively increased the catalytic performance of Ir/MoC. This work offers guidelines for creating stable and effective CO_2_ utilization catalysts.

With In_2_O_3_, Mhamane’s work [81] showed a sustainable catalytic CO_2_ conversion to almost 100% CO selectivity at room pressure. Using less hydrogen in the feed than stoichiometric amounts, it is important to note that high CO yield may be observed at the expense of unwanted methanation; 1:1 and 1:0.67 CO_2_:H_2_ ratios show 98–99.6% CO selectivity with 25–38% CO_2_ conversion between 773 and 873 K. For the reverse water–gas shift reaction, an ideal reactant composition is present when CO_2_ and H_2_ conversion occurs under steady-state conditions at 773–873 K, suggesting a 1:1 ratio of adsorbed reactants (with 1:0.67 CO_2_:H_2_ feed) on the catalyst surface. In contrast, H_2_–rich feed compositions indicate the H_2_–dominated surface. Using near-ambient pressure photoelectron spectroscopy (NAPPES) to investigate surface electronic structure changes under near–operating conditions, the following intriguing results were found: (a) Higher temperatures caused a shift in the valence band to lower binding energy, up to 0.6 eV. (b) Active oxygen vacancy site formation modifies the In_2_O_3_ work function and is responsible for the catalyst surface’s observed heterogeneous nature under NAPPES measurement conditions. (c) It is discovered that the aforementioned alterations are reversible upon cessation of the reaction. We found that in the active temperature window of the catalyst supporting the heterogeneous reaction, the vibrational characteristics of the reactant molecules were widened.

Precious metal catalysts (Pt, Pd, Au, Rh, Ru, Ir, etc.) are not easy to deactivate, although they have high catalytic activity and stability. However, due to their high prices and scarce resources, they are limited in large–scale applications in industrialization and are only suitable for laboratory mechanism research. Therefore, efforts are needed to find some alternative catalysts to precious metals.

### 2.4. Non–Precious Metal Catalysts

Although precious metal catalysts have excellent CO_2_ reduction performance and stability, their high cost limits their large−scale industrial application. Therefore, the exploration of inorganic non-precious metal catalysts has attracted widespread attention from scientific research staff, and non-precious metal catalysts (Cu, Ni, Fe, Mo, etc.) are often used to replace precious metals because of their low cost and good catalytic activity.

#### 2.4.1. Ni–Based Catalysts

Nickel-based catalysts experience the problems of easy sintering and carbon deposition in the RWGS reaction and have low selectivity for CO. Several examples of design, preparation, and mechanism research on nickel-based catalysts are listed below.

Zhang et al. [48] prepared a sulfur-containing Ni/ZrO_2_ catalyst to improve CO_2_ conversion and regulate CO_2_ hydrogenation selectivity. The effect of carrier size on the catalytic performance of RWGS was investigated. The results show that the 80 nm Ni/ZrO_2_ sample with a smaller carrier size had higher Ni dispersion and oxygen-vacancy concentration, had more exposed active centers, and displayed improved adsorption and activation ability of CO_2_.

Gong et al. [82] proved that the interfacial synergism of Ni/Ga_2_O_3_ promoted the selective hydrogenation of CO_2_ to CO (CO selectivity > 95%) in the temperature range of 350–450 °C. Studies show that the synergistic effect of the Ni/Ga_2_O_3_ interface significantly affects the adsorption of H_2_ and CO_2_, resulting in the formation of different intermediates and products. *HCOO preferentially forms on the Ni surface and is further hydrogenated to CH_4_ and H_2_O. In contrast, the Ni/Ga_2_O_3_ interface favors the formation of CO with the help of H_2_. This study provides a highly selective catalyst for the RWGS reaction in the CO_2_ hydrogenation process and promotes the surface modification of the catalyst to improve the activity and selectivity.

Our group [8] prepared NiCe−HMS and Ni-HMS limited-structure catalysts using the one-pot method and conducted RWGS tests in the 500–750 °C temperature range. Compared with Ni/HMS catalysts prepared via the traditional impregnation method, Ni−HMS and NiCe−HMS showed higher CO selectivity. This is due to the formation of highly dispersed nickel nanoparticles on the surface of the carrier, which inhibits the formation of CH_4_.

Zhang et al. [83] reported the activity and selectivity of Ni-rich nickel phosphide catalysts for CO_2_ hydrogenation for the first time. Their findings demonstrate that Ni_2_P/SiO_2_ is an extremely effective catalyst for RWGS, yielding more than 80% of CO throughout the entire 350–750 °C temperature range.

Shen et al. [84] discovered that Ni doping (1.0−Ni−CeO) significantly increased the RWGS reaction activity of pure CeO_2_ at low temperatures. At 300 °C and a WHSV of 60,000 mL·g^−1^·h^−1^, the Ni−based mass−specific CO formation rate of 1.0−Ni−CeO_2_ could reach up to 1542 mmol·g_Ni_^−1^·h^−1^. Furthermore, it was established through our experiments and density functional theory calculations that the surface defects serve as the active sites for H_2_ heterolytic dissociation, which is the RWGS reaction’s rate-determining step. In 1.0−Ni−CeO_2_, the formed Ce−H species is stable. Furthermore, the superior H_2_ heterolytic dissociation capacity and high active H^−^ released from the Ce-H species of 1.0−Ni−CeO_2_ at low temperatures are responsible for its increased catalytic activity and selectivity. Moreover, CO−TPD (temperature−programmed desorption) and CO DRIFTS demonstrated that 1.0−Ni−CeO’s 100% CO selectivity was primarily due to its lower CO affinity. A new reaction mechanism was revealed using systematic in situ DRIFTS analysis. According to this mechanism, CO_2_ was first adsorbed on the hydroxyl groups of Ce^3+^−OH rather than on oxygen vacancies, which resulted in the formation of the bicarbonate intermediate (*HCO_3_). The bicarbonate intermediate was then reduced to formate (*HCOO), and the highly active H^−^ in Ce−H caused the production of CO. This work may open up new avenues for the development of robust, affordable, and efficient catalysts for hydrogenation reactions at low temperatures.

In addition, many other nickel catalysts have been reported [48,49,85,86,87,88,89,90,91,92,93,94,95,96,97,98], including bimetallic [93,94,95,96,97,98] or polymetallic catalysts composed of nickel and other metals.

#### 2.4.2. Fe−Based Catalysts

Fe−based catalysts [50,99,100,101,102,103,104,105], as typical high-temperature catalysts, have also been used in RWGS reaction studies. Here are some selected examples. The carrier−free nano ferric oxide catalyst prepared by Kim et al. has high catalytic activity and stability [50]. Through transmission electron microscope observation, it was found that nano iron oxide did not easily agglomerate during the RWGS reaction. XPS and X–ray diffraction (XRD) show that atomic carbon and oxygen formed due to dissociative chemisorption of CO or CO_2_ appear to diffuse into many nanoparticles to form Fe oxides and Fe carbides. C and O are produced by the reactants on the surface of the iron oxide, and CO_2_ and CO are diffused into the body of the iron oxide nanocatalyst. Therefore, during the RWGS reaction, the structure of the catalytically active surface consisting of metal Fe remains unchanged, so the catalytic activity remains stable for a long time. For Fe–based catalysts, it is still a subject of debate as to whether the active phase is metallic iron, iron oxide, or iron carbide.

Bogdan et al. [99] investigated, for the first time, the reduction of CO_2_ to CO with hydrogen on alumina–supported Co and Fe catalysts under supercritical conditions with CO or CH_4_ as the target product. The selectivity of the Co/Al_2_O_3_ catalyst for methanation is close to 100%, while the Fe/Al_2_O_3_ system shows an advantage for hydrogenating CO_2_ to CO and significantly forms ethane (up to 15%). Compared with the gas–phase reaction, the spatio–temporal yield can be increased by one order of magnitude under supercritical conditions. The difference in the crystal phase characteristics of the iron-containing catalyst leads to the reverse water–gas transfer reaction to produce carbon monoxide, while the reduced iron phase leads to the Fischer–Tropsch reaction to produce a mixture of hydrocarbons. Direct methanation occurs selectively on Co catalysts. Methanol formation was not observed on the studied Fe and Co catalysts.

Zhang et al. [100] found that metallic iron was a better RWGS catalyst than Fe_3_C. The addition of Cs and Cu can promote the activity of Fe^0^, hindering the carburization of iron while favoring higher conversion rates and selectivity. When stability tests were performed, the catalyst aged during the RWGS reaction, and a new phase appeared: Fe_5_C_2_ (Hagg carbide). For RWGS reactions, Fe_5_C_2_ is an excellent catalyst with a higher carbon dioxide conversion rate than samples in which Fe⁰ is the active phase. However, Fe_5_C_2_ has a low CO selectivity compared to Fe^0^-based samples. The results showed that the best activity/selectivity was achieved by fine-tuning the Fe/Fe₅C_2_ ratio.

Watanabe et al. [101] used H_2_S as a co–feeding gas to investigate the catalytic activity of Fe/CeO_2_ on the RWGS reaction and maintained a stable activity for 12 h. In addition, co–feed H_2_S can be used as a hydrogen source for RWGS.

In Xu’s study [102], the comparison of dynamic and steady−state CO/CO_2_ hydrogenation performance showed a large amount of FeO_x_ covering the χ−Fe_5_C_2_−dominated bulk phase during CO_2_ hydrogenation. The overall RWGS rate was positively correlated with the surface content of FeO_x_ under differential conditions (CO_2_ conversion < 10%) and negatively correlated with the surface content of FeO_x_ under integral conditions (CO_2_ conversion > 10%).

Their findings help us to understand the role of different types and amounts of iron sites on Fe–based catalysts during CO_2_ hydrogenation. Adjusting the number and properties of different surface sites to achieve optimal intersite synergies may stimulate the development of future multi−site CO_2_ hydrogenation catalysts.

#### 2.4.3. Cu-Based Catalysts

Cu-based catalysts have attracted much attention because of their low price, high activity at relatively low temperatures, and good selectivity to CO. However, since copper is easily sintered or coked, such catalysts are prone to deactivation during the RWGS reaction. In order to overcome these shortcomings, many RGWS reactions catalyzed by Cu–based catalysts have been reported [39,51,52,53,106,107,108,109,110,111,112,113,114,115,116,117,118,119,120,121,122,123,124,125,126,127,128,129,130,131], and we will describe some representative examples in detail.

Ai et al. [106] discovered that the grinding process could enhance the amount and distribution of Cu^0^ species on the surface and that the Cu content could control the amount and distribution of Cu^0^ species’ crystallinity. Moreover, the low crystallinity of Cu^0^ species could encourage the exposure of CO_2_–hydrogenation active sites.

Zhuang et al. [51] added Ru to the Cu/ZnO/Al_2_O_3_ catalyst for the RWGS reaction. The CO_2_ conversion rate was more than two times higher than that of the Cu/ZnO/Al_2_O_3_ catalyst. At the same time, the stability of the catalyst was significantly improved. Through XRD, scanning electron microscope–energy−dispersive spectroscopy (SEM–EDS), scanning transmission electron microscope–energy–dispersive spectroscopy (STEM–EDS), and TPR characterization, it was found that this may be due to the formation of Ru–Cu core–shell nanoparticles. In addition, the interaction between Ru and the support is also an important factor affecting catalyst selectivity.

Liu et al. [38] constructed stable copper clusters in a Cu/CeO_2_ catalyst with a high copper loading capacity of 15 wt%. Under very harsh reaction conditions, CeO_2_ nanorods were partially sintered, forming 2D and 3D copper clusters on their surfaces. This partially sintered catalyst exhibits unparalleled activity and excellent durability at high temperatures. The interaction between copper and CeO_2_ ensures that the copper clusters are stably anchored to the CeO_2_ surface. The large number of in situ surface-oxygen vacancies causes a synergistic effect with adjacent copper clusters, which promotes the reaction.

Deng et al. [39] prepared catalysts with different copper loads using the simple impregnation method via NaOH−activated slag-polymer microspheres (SGSs) as the carriers. The results show that the 10% Cu/SGS catalyst has better CO_2_ conversion performance. At 550 °C, the CO_2_ conversion and CO selectivity of the 10% Cu/SGS catalyst reached 48% and 96%, respectively. The results show that the high performance of the catalyst is mainly due to the interaction between copper and the SGS carriers and the adsorption and activation of CO_2_ by the alkaline center on the SGS carriers.

Liu et al. [107] loaded Cu onto titanium dioxide nanotubes (TiNT) and titanium dioxide nanoparticles (TiNP), and the RWGS activities of the two catalysts were different. The activity of the nanoparticle carrier is very low, and the content of Cu hardly changes the activity, but the activity of the nanotube carrier is very high and has three different behaviors as the surface density of copper increases. At a low surface density, an active Cu−O−Ti site with low apparent activation energy is formed. At a higher surface density, copper nanoparticles are formed on the surface of TiNT, and the reaction barrier is reduced. At moderate surface densities, the metal copper clusters are engulfed by a TiO_x_ overlay formed during hydrogen treatment, similar to the overlay formed by classical strong metal carrier interactions (SMSI). These reducing layers are significantly more active against RWGS than the initial TiNT surface but have similar activation barriers, higher than those on exposed copper and TiNP surfaces. SMSI is an important concept in heterogeneous catalysis, but it will inevitably sacrifice catalytic activity due to over-coverage.

González–Arias et al. [108] prepared a series of Cu−MnO_x_ catalysts and found that the improvement in catalyst performance was due to the addition of Mn to enhance the dispersion of Cu and increase the surface alkali concentration. Under standard RWGS conditions, the highest reaction rate of the catalyst is related to the improvement of Cu dispersion and the composition of the highly active Cu−MnO_x_ domains. It is worth noting that the change in the optimal copper–manganese ratio is a function of the RWGS reaction conditions.

Ebrahimi et al. [52] prepared a Cu/CeO_2_ solid solution using the combustion synthesis method. The produced catalyst exhibited high activity, stability, and 100% CO selectivity in the RWGS reaction, and these catalytic activities were attributed to the excess oxygen vacancy on the CeO_2_ surface, which could promote CO_2_ conversion. The high activity of this catalyst is due to the synergistic effect between the active Cu^0^ and Ce^3+^−oxygen vacancy.

**Figure 4:**
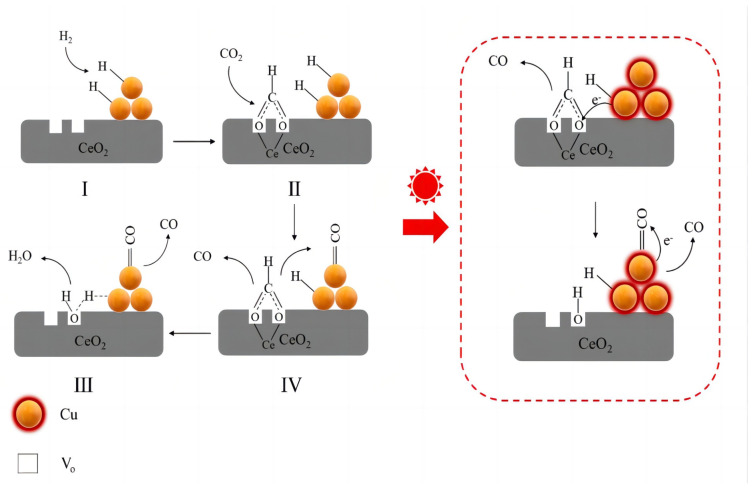
The proposed mechanism of the RWGS reaction under visible−light irradiation.

Yang et al. [109] reported the reduction reaction of carbon dioxide catalyzed by Cu−CeO_2_ under visible light and speculated regarding a reaction mechanism induced by the localized surface plasmon resonance effect with the assistance of visible light. The specific reaction process is divided into four steps [Figure 4]: (I) hot electrons and hot holes are produced via the plasmon effect of Cu when visible light is present; (II) hydrogen dissociation and spillover are accelerated via the hot holes’ reaction with hydrogen; (III) CO is produced via the hot electrons attacking and transferring to the intermediate species; and (IV) vibrant light exposure causes the oxygen vacancies to regenerate.

Zhang et al. [53] studied a variety of strategies to effectively regulate nanointerfaces from different aspects, such as carrier composition, Cu preparation parameters, and pretreatment and reaction conditions, in order to balance the activity and stability of Cu/TiO_2_ catalysts in RWGS reactions. The results show that due to the reducibility of TiO_2_ and the limited electron transfer from TiO_2_ to Cu, only doping Zn in the TiO_2_ carrier can inhibit the formation of nanointerfaces during the reduction treatment. Cu coverage on Zn−modified TiO_2_ is significantly decreased, but the catalytic activity is increased by 44%, and the stability is unchanged.

In addition to the specific examples mentioned above, many other researchers have reported interesting works [39,53,110,111,112,113,114,115,116,117,118,119,120,121,122,123,124,125,126,127,128,129,130,131] on copper catalysts, mainly focusing on catalyst design and mechanism studies.

#### 2.4.4. Mo-Based Catalysts

Molybdenum is more abundant and cheaper than precious metals, increasing the potential for large−scale industrial applications of molybdenum−based catalysts. Molybdenum−containing catalysts are widely used, including Ni−Mo catalysts [132], molybdenum sulfide catalysts [133], MoO_x_ catalysts [54], supported Mo-based catalysts [55,134], new Mo_2_C and Mo_2_N catalysts [135], etc. A few representative examples are described below.

Xin et al. [136] found that during the CO_2_ hydrogenation reaction, Ru−MoO_3_ formed a strong metal−support interaction (SMSI) state between the metal and the carrier at 250 °C, which was conducive to CO_2_ selective hydrogenation to form CO. During the reaction, Ru nanoparticles promote the reduction of MoO_3_ to form an active MoO_3−x_ coating with oxygen vacancies, which migrates to the surface of Ru nanoparticles to form a coating structure (Ru@MoO_3−x_). The resulting SMSI state changes the catalytic performance of the catalyst from 100% methanation at the Ru site on the exposed surface to 99% CO formation via the quasi-MvK (Mars–van Krevelen) mechanism in the MoO_3−x_ layer. Selective regulation is achieved through different SMSI states in the CO_2_ hydrogenation reaction.

Ronda [56] et al. took advantage of the high thermal stability and excellent electrical conductivity of the MAX phase to prepare a MoO_3_/Ti_3_AlC_2_ catalyst to increase its intrinsic RWGS activity. When molybdenum oxide is loaded on the MAX phase of the Ti_3_AlC_2_, the low surface area of the MAX phase leads to the formation of large MoO_3_ rods with blocky characteristics. The presence of electron-rich Ti_3_AlC_2_ enhanced the redox characteristics of MoO_3_ in the RWGS reaction, resulting in a high degree of surface reduction and the formation of many oxygen vacancies. Therefore, the catalyst showed the highest activity in catalytic experiments. When the MAX phase acts as a carrier, the electron-rich Mo site is an ideal activation site for CO_2_ via electron transfer to the CO_2_ antibond orbital, an interaction that weakens the C−O bond and favors reduction to CO. The catalyst is selective to CO, thus inhibiting the formation of methane and coke.

Zhang et al. [54] found that the surface modification of Ni by MoOx can regulate RWGS and methanation reactions. The addition of MoO_x_ improves the dispersion of Ni through strong interaction, and the partially reduced MoO_x_ modifies the surface of Ni particles using covering and electron modification, which enhances the desorption of CO on the surface. The addition of a large amount of Mo (Mo/Ni ratio of 1) causes the reaction to shift to RWGS and the CO selectivity to be greater than 94%. Kharaji et al. [57] incorporated Mo into the Fe/Al_2_O_3_ catalyst and found that Mo greatly improved the RWGS catalytic activity and CO selectivity due to the Fe−O−Mo structure formed in the Fe_2_(MoO_4_)_3_ phase of the Fe−Mo/Al_2_O_3_ catalyst. The presence of the Fe−O−Mo structure causes Fe_2_(MoO_4_)_3_ to have a lower reducibility, which effectively inhibits the reduction in Fe oxides. On the other hand, the existence of the Fe–O–Mo structure promotes the flow of electrons from Fe to Mo, resulting in the Fe species being in an electron–deficient state and forming a positive surface charge, which is detrimental to the adsorption of CO and effectively inhibiting the further hydrogenation of CO. As an additive and active component, Mo shows good CO selectivity, but its CO_2_ conversion needs to be improved.

Zhang et al. [137] reported the preparation of Cu−Cs−MO_2_C using Cu as an accelerator and Cs as a dopant to improve the conversion and selectivity of the catalyst, respectively. It was found that the increase in CO_2_ conversion was due to the addition of copper to provide more active sites for the catalyst, such as Cu^+^ and Cu^0^. Cs was due to its significant positive electric properties that create electron perturbations on the catalyst surface, thus enhancing catalytic performance. Its addition can improve CO selectivity, especially at low temperatures. In addition, the carbon–doped catalyst appears to be activated in situ due to the recarburizing phenomenon, which results in the catalyst being fairly stable in continuous operation.

Zhang et al. [138] used silica as the carrier to prepare a Mo−P−SiO_2_ catalyst and found that the catalyst was completely oxidized through characterization. It was found that the high selectivity of carbon monoxide was due to the MoP phase generated on the surface of the silica carrier, which was more conducive to the direct conversion of carbon dioxide into carbon monoxide through MoP(0001). Based on the potential energy−surface profile, methane generation on the MoP(0001) surface requires higher energy than carbon monoxide. In other words, the MoP(0001) surface is more selective for producing CO than CH_4_.

Yuan et al. [139] reported the application of Ni−doped MoS_2_ in CO_2_ hydrogenation into methanol and prepared a series of MoS_2_/Ni_X_ catalysts. Through DFT calculations, they found that MoS_2_/Ni–catalyzed CO_2_ hydrogenation follows the oxidation–reduction pathway, and the optimal adsorption structures and barrier energies of different intermediates were shown in the article. CO_2_ and H_2_ are directly dissociated from CO*, O*, and 2H* by an energy barrier of 0.63 eV. The large adsorption energy indicates that CO* has a strong interaction with MoS_2_ (Eads = −1.27 eV) and is more likely to react further on the carrier without releasing CO.

### 2.5. Other Catalytic Systems

#### 2.5.1. Transition Metal Carbide Catalysts

Transition metal carbide catalysts (TMCs) are also promising and attractive catalysts; after the introduction of carbon, the electronic properties of these catalysts change, meaning that their activity becomes similar to that of precious metal catalysts, with a strong H_2_ dissociation and CO bond-breaking ability, and showing high activity in the CO_2_ hydrogenation to CO reaction. Therefore, it has been reported that catalysts such as vanadium carbide [140], tungsten carbide [141,142], cobalt carbide [143], and molybdenum carbide [126,144,145,146,147,148,149] have superior catalytic activity and CO selectivity when applied to RWGS reactions.

Juneau et al. reported K-promoted tungsten carbide with porous *γ*−Al_2_O_3_ as the carrier for RWGS in 2019 [141]. Later, they [142] found that micellar-based silica coating technology could prepare a 10 nm tungsten carbide nanoparticle catalyst. The high activity and CO selectivity of nano-WXC carburizing observed at 1000 °C may be due to the fact that the coated silica maintains a particle size of 10 nm during carburizing, thus promoting the formation of surface carbides. Compared with the catalyst prepared via the impregnation method, the synthesis method affects the formation degree of W × C and the reactivity of RWGS. In Zhang’s [143] paper, they described a mechanism for controlling the formation and quantity of carboxylate species on hollow cubic Co_3_O_4_ (without Mn), thereby modifying the surface energy and crystal growth rate. At 270 °C, where CO_2_ conversion was almost at its thermodynamic limits at a space velocity of 60,000 mL g_cat_^–1^ h^–1^, Co_2_C nanoprisms demonstrated excellent activity in RWGS. Reaction mechanisms and kinetics studies were used to correlate the catalytic performance of Co_2_C nanoprisms with highly active surfaces (020) and (101), as well as double reaction pathways (redox and formate routes). This work shows great potential for bridging RWGS and sequential cascade reactions, and it offers a way to design and modulate the morphologies of transition metal carbides. Reddy [144] synthesized Mo_2_C using the direct carbonization of Mo precursors. Under 723 K and 1 atmospheric pressure with a molar ratio of CO_2_/H_2_ = 1:3, the conversion of CO_2_ catalyzed by Mo_2_C can reach 57%, and the CO selectivity can reach 62%. Marquart et al. [145] prepared four catalysts using three different synthesis processes with good catalytic activity. The results show that CO_2_ forms adsorbed oxygen surface species by dissociating on the carbide surface. Above 850 K, the carbides oxidize into the oxide phase MoO_2_ or MoO_3_. The different catalysts all exhibit CO_2_ conversion rates higher than 30%, and the CO selectivity is 99% even at high H_2_/CO_2_ ratios. Although the new molybdenum carbide catalyst has excellent activity in the RWGS reaction, its preparation method is difficult compared with other catalysts.

#### 2.5.2. Perovskite–Type Catalysts

Mixed metal oxides with ABO_3_-type perovskite structures acting as carriers of rare earth metals and transition metals are considered to be very promising catalytic ceramic materials [150]. The catalytic properties of perovskites can be modulated by changing the properties of A and B ions or inserting dopants into the structure. Perovskite oxides have attracted the attention of researchers due to their high mobility of oxygen and ability to stabilize unusual cationic oxidation states, as well as their thermal stability at high temperatures [151]. Alkali metals, alkaline earth metals, and rare earth metals are commonly used as accelerators, which can change the acid–base properties of the catalyst, improve the dispersion of the active species, and increase the strength of the interaction between the active species and the carrier. Kim et al. [152] added Y, Zn, and Ce to BaZrO_3_ and discussed the influence of dopants on the catalytic activity. At 600 °C for 5 h, all catalysts showed stable catalytic properties. Among them, the BaZr_0.8_Y_0.16_Zn_0.04_O_3_ (BZYZ) catalyst showed excellent activity with a CO_2_ conversion rate of 37.5% and CO selectivity of 97%. Ce insertion improved the ionic conductivity of BZYZ, but additional Ce did not have any positive effect on the catalytic activity of the RWGS reaction. Jo et al. [153] examined the effects of controlled template removal and nanocasting on La_0.8_Sr_0.2_FeO_3_ (LSF). The nano−cast LSF has a mesoporous structure due to using the SBA−15 template, and further etching enhances its textural characteristics. Characterization verified the high dispersion of metal–active sites on nano–cast LSF. RWGS operating at 600 °C used LSF as oxygen carriers with varying silica contents. It was discovered that the CO yield rose as the silica residue in LSF decreased; however, the catalyst’s mesoporous structure would be destroyed by excessive etching, removing too much silica (<5 wt%). An LSF with a 10 wt% silicon content outperforms RWGS. The surface−oxygen−to−lattice−oxygen atomic ratio could be a crucial factor in determining the reaction’s reactivity. The photothermal catalyst Ni/LaInO_3_ designed by Yu et al. [154] can convert CO_2_ into nearly uniform CO in a H_2_−rich environment with a high generation rate, which promotes the adsorption and activation of CO_2_. Due to the photoelectric effect, the RWGS reaction time can be significantly reduced. This study provides a new idea for designing efficient photothermal catalysts to maximize the use of solar energy in the future and lays the foundations for regulating product selectivity during catalytic CO_2_ hydrogenation.

In heterogeneous catalysis, the catalytic performance of bifunctional catalysts is often better than that of single-component catalysts. The shape, size, and distribution of catalytic particles also have a significant influence on the activity of the catalyst. Kopac et al. [155] theoretically studied the effects of bifunctional groups on RWGS reactions in detail. Density functional theory was used to calculate the energy diagram of RWGS on three surfaces (copper (111), SrTiO_3_ surface, and Cu/SrTiO_3_ interface between two solids). These three surfaces were combined using mesoscale dynamics Monte Carlo simulations to study the turnover and yield of CO production as a function of particle size. The results show that the reaction speed at the interface is faster. However, in addition to the interface, the binding of copper and carrier sites further accelerates the RWGS reaction, suggesting that the catalyst’s bifunction is manifested in more complex interactions between phases rather than just interface effects, such as hydrogen spillover. The authors found three different effects, namely an electronic effect, a synergistic effect, and a geometric effect, and a smaller Cu on the carrier SrTiO_3_ showed a higher CO formation rate.

### 2.6. Catalyst Deactivation in the Reverse Water–Gas Conversion Reaction

Under high-temperature conditions, the catalyst used for RWGS reaction has poor thermal stability or weak interactions between the active component and the carrier, which leads to sintering and agglomeration of the catalyst at high temperatures and decreases the activity of the catalyst [58]. In addition, carbon deposition is also an important cause of catalyst deactivation. Carbon deposition, as the name suggests, means that the surface of the catalyst is covered by a certain form of carbon, and carbon is mainly formed via carbon-containing substances in the raw material breaking bonds on the surface of the catalyst or first coking and then dehydrogenation. These carbon species cover the active site, thus reducing catalyst activity. Therefore, the stability of the catalyst at high temperatures is extremely important for the RWGS reaction.

Yang et al. [58] mixed Fe into Ni/CeO_2_–Al_2_O_3_, and the NiFe/CeO_2_–Al_2_O_3_ catalyst showed an excellent CO_2_ conversion rate in the stability test at 800,000 mL·g^1^·h^−1^ and 700 °C. FeO_x_ greatly enhances Ni dispersion on the surface, which helps to provide higher activity in the reaction. In addition, the FeO_x_–Ni interaction leads to electron enrichment of Ni surface atoms, and higher electron density is conducive to CO_2_ adsorption. The 0.3CuMgAl–LDH–400 catalyst prepared using the hydrothermal method by Chen et al. [156] showed high stability during the RWGS reaction, and the activity remained unchanged for more than 30 h, which was due to the fact that the use of LDH as a carrier improved the dispersion of Cu and the presence of more alkaline sites. On the contrary, the catalytic activity of 0.3CuMgAl–IMP–400 prepared using impregnation decreased slightly after 20 h of the reaction. The results show that the catalyst prepared with LDH as the support has better stability.

Goguet et al. [59,157] studied the deactivation process of Pt/CeO_2_ catalysts. The stability test results show that the initial CO_2_ conversion rate is stable at 13.7%, and the CO selectivity is greater than 99%. As the reaction progresses, its catalytic activity begins to decrease gradually, possibly due to the catalyst’s carbon deposition or the metal’s sintering. In order to clarify the deactivation principle, the TPO cycle test was performed on the Pt/CeO_2_ catalyst before and after the reaction. The results show a linear relationship between the degree of deactivation of the Pt/CeO_2_ catalyst and the amount of carbon deposition. The place where carbon deposition occurs is the active part of the reaction. With the increase in time, the active part is gradually covered by carbon deposition, which leads to catalyst deactivation.

## 3. Conclusions

Converting carbon dioxide into high-value-added chemicals and fuels is a potential path to a carbon-neutral future. The reverse water–gas conversion reaction converts carbon dioxide into carbon monoxide and produces syngas, followed by methanation and the preparation of alcohols using Fischer–Tropsch synthesis, which is a promising method for carbon dioxide utilization. However, due to the endothermic reaction at low temperatures, the strong exothermic methanation reaction is competitive with the reverse water–gas change reaction. Although a high temperature is conducive to the forward reaction, the energy consumption of the reaction is too high. Therefore, developing catalysts capable of high conversion and selectivity at lower temperatures in the future will be a research focus for scientists. Transition metals ought to be the main subject of future research on the elemental makeup of active RWGS catalysts. Future resource shortages are inevitable, but they can be minimized by avoiding reliance on noble and rare elements. Targeting less expensive components will also lower overall expenses. Common elements like Cu, Fe, and Mo are important for further RWGS research because they can significantly increase catalytic activity, particularly when combined with metal oxides as a support. Future theoretical and computational investigations, in addition to experimental catalytic testing (i.e., DFT and general kinetic models), are required to facilitate the preparation of catalysts and to investigate the energetics of intermediates at various points within the catalytic structure through mechanism studies. Throughout the catalytic process, the catalyst’s deactivation and stability will be taken into account. Finally, for the development of catalysts with high CO_2_ conversion rates and high CO selectivity, we can proceed based on the following aspects: (1) improving the preparation method for catalysts to improve the dispersion of active components and (2) modifying the carrier to increase the dispersion of metal species and adjust the interaction between the active component and the carrier. In addition, we may be able to promote the development of low-carbon industrial catalysis using light-driven chemical reactions and develop and design suitable catalysts to gradually transition RWGS reactions from thermal reactions to photocatalytic processes.

## Figures and Tables

**Table 1:** A brief summary of the RWGS reaction conditions of selected catalysts and their CO_2_ conversion and CO selectivity.

Catalyst	Reaction Gas Ratio, H_2_/CO_2_/Inert Gas	Temperature °C	Reaction Airspeed mL·gcat^−1^mL·h^−1^	CO_2_ Conversion Rate %	CO Selectivity %
Cu/CeO_2_ [37]	3:1:0	600 °C	400,000	50	100
Cu/SGS [38]	4:1:0	550 °C	3600	48	96
Pt/CeO_2_ [40]	9:9:2	500 °C	30,000	30	-
Pt_cluster_/PN−CeO_2_ [41]	3:1:0	300 °C	12,000	17.5	99.9
Pt_1_/SiC [32]	1:1:0	900 °C	-	54	100
Pt−Re/SiO_2_ [42]	4:1:5	400 °C	60,000	24.3	96.2
Pt/SiO_2_ [43]	1:4:5	400 °C	60,000	12.1	100
CoPd-CoO_OV_ [44]	3:1:0	300 °C	-	-	94
Ni/ZrO_2_ [45]	4:1:4	500 °C	13,500	27.6	100
Re_2_O_7_/SZ [46]	4:1:0	400 °C	-	18	95
Ni/Ga_2_O_2_ [47]	4:1:5	450 °C	60,000	40	95
Fe−oxide [48]	1:1:0	600 °C	24,000	38	>85
Ni−MgO−Ce_0.8_Zr_0.2_O_2_ [49]	63:21:16	250 °C	50,000	4.5	90.5
Cs/Fe,O [50]	4:1:0	450 °C	12,000	58	75
Cu/MnO_x_ [51]	12:3:5	550 °C	60,000	55.5	100
1 wt.%Cu−CeO_2_ [52]	4:1:0	600 °C	-	70	100
MoO_3_/FAU [53]	12.5:12.5:10	500 °C	7500	15	100
Ru@MoO_3-x_ [54]	9:3:88	250 °C	100,000	45	>99
MoO_3_/TiAlC_2_ [55]	4:1:0	550 °C	15,000	30	-
Cu−Cs−Mo_2_C [56]	4:1:0	600 °C	12,000	-	100
Mo−P−Si_2_O [57]	4:1:0	550 °C	12,000	18	100
Ni/CeO_2_−Al_2_O_3_ [58]	4:1:0	750 °C	30,000	59	94
2%Pt−CeO_2_ [59]	4:1:0	290 °C	-	27.1	100

## Data Availability

Data are contained within the review.
